# Simultaneous manipulation of multiple genes within a same regulatory stage for iterative evolution of *Trichoderma reesei*

**DOI:** 10.1186/s13068-022-02122-0

**Published:** 2022-03-05

**Authors:** Xianhua Sun, Yazhe Liang, Yuan Wang, Honglian Zhang, Tong Zhao, Bin Yao, Huiying Luo, Huoqing Huang, Xiaoyun Su

**Affiliations:** 1 State Key Laboratory of Animal Nutrition, Institute of Animal Sciences, Chinese Academy of Agricultural Sciences, Beijing, 100193 China; 2 Institute of Microbiology, Chinese Academy of Sciences, Beijing, 100101 China

**Keywords:** *Trichoderma reesei*, Semi-rational, Cellulase, High-throughput, Automation, Biofuel

## Abstract

**Background:**

While there is growing interest in developing non-canonical filamentous fungi as hosts for producing secretory proteins, genetic engineering of filamentous fungi for improved expression often relies heavily on the understanding of regulatory mechanisms.

**Results:**

In this study, using the cellulase-producing filamentous fungus *Trichoderma reesei* as a model system, we designed a semi-rational strategy by arbitrarily dividing the regulation of cellulase production into three main stages-transcription, secretion, and cell metabolism. Selected regulatory or functional genes that had been experimentally verified or predicted to enhance cellulase production were overexpressed using strong inducible or constitutive promoters, while those that would inhibit cellulase production were repressed via RNAi-mediated gene silencing. A *T. reesei* strain expressing the surface-displayed DsRed fluorescent protein was used as the recipient strain. After three consecutive rounds of engineering, the cellulase activity increased to up to 4.35-fold and the protein concentration increased to up to 2.97-fold in the genetically modified strain.

**Conclusions:**

We demonstrated that, as a proof-of-concept, selected regulatory or functional genes within an arbitrarily defined stage could be pooled to stimulate secretory cellulase production, and moreover, this method could be iteratively used for further improvement. This method is semi-rational and can essentially be used in filamentous fungi with little regulatory information.

**Supplementary Information:**

The online version contains supplementary material available at 10.1186/s13068-022-02122-0.

## Background

The filamentous fungi workhorses *Trichoderma reesei*, *Aspergillus niger*, and *Aspergillus oryzae* are well-known for their prominent ability to produce secretory proteins. In addition to these commonly used fungal hosts, there is growing interest in developing non-canonical filamentous fungi such as *Humicola insolens* [1] and *Myceliophthora thermophila* [2] as protein producing cell factories. These fungi are unique in their specialized ability to produce enzymes with intriguing properties, e.g., neutral cellulase from *H. insolens* [3] and thermophilic cellulase from *M. thermophila* [4]. In *T. reesei* and *Aspergillus* spp., many molecular mechanisms governing the gene expression and protein secretion have been deciphered. In contrast, for the former filamentous fungi, the mechanisms underlying protein expression and secretion are poorly known.

Both the prevalent and non-canonical filamentous fungi can be improved by random mutagenesis, which normally introduces many genetic changes to the chromosomes. This is manifested in the cellulase hyperproducer *T. reesei* RUT-C30 strain: during mutagenesis, the fungus lost a chromosomal region of 85 kb (29 gene-encoding) among the other mutations [5]. Although the contribution of each mutation to the enhanced cellulase-producing ability has not been clearly defined, it is clear that the improvement is a consequence of cumulative benefits from multiple mutations including the truncation of the transcription repressor *cre1* [6] and a frameshift mutation in the glucosidase II involved in glycosylation [7]. Compared with random mutagenesis, rational genetic engineering provides more precise control of protein production. By both means, manipulation of multiple genes, but no single one, is essential in the success towards developing a protein hyperproducer. For example, a mutant strain of RUT-C30 could produce 134% improved endoglucanase activity. This mutant was generated by integrating two mutations including overexpressing *xyr1* (the master transcription activator) and RNAi-mediated gene silencing of *ace1* (a transcription repressor)[8]. Despite the advantage of saving time and labor, however, this strategy cannot be used for the fungi with little information about molecular mechanisms regulating secretory protein production.

The past decades have witnessed the successes in genome sequencing of many filamentous fungi as well as in introducing multiple genes into a single filamentous fungal cell such as *T. reesei* [9], *Trichoderma guizhouense* [10], and *M. thermophila* [11]. The availability of sequenced genomes suggests that uncharacterized homologs of known regulatory genes in the prevalent fungi might play similar, orthologous roles in non-canonical filamentous fungi. Additionally, the success in simultaneous transformation of a selected pool of functional genes raised a possibility to manipulate those whose expression impacts secretory protein production. A combination of these two means might serve as an alternative strategy of rational genetic engineering and be particularly useful in filamentous fungi with little knowledge about the regulatory mechanisms. In this way, the possibility of introducing the true effecting regulator(s) could be increased. Additionally, due to the undefined copy numbers and insertion loci in filamentous fungi, combining multiple, but not one, genes in one transformation will create rich diversity in the expression profiles of the targeted genes in the transformants. Therefore, the plethora of expression profiles of the targeted genes would be able to further raise the possibility to generate protein hyperproducers and accelerate the process of strain improvement. This design is essentially semi-rational.

Simultaneous use of pooled functional genes has been successfully used in bacteria [12] and unicellular eukaryotic cells such as *S. cerevisiae* [13]. However, transformation of pooled functional genes has been seldom reported in filamentous fungi. To demonstrate for proof-of-concept if the semi-rational engineering strategy can be used in filamentous fungi, we chose *T. reesei* as a model system and tested the strategy to improve its ability to secret cellulase. *T. reesei* is one of the most important industrial filamentous fungi due to its prominent ability to secrete cellulase as well as heterologous proteins [14]. In *T. reesei*, production of cellulase is affected by several processes involving multiple regulatory stages. We arbitrarily divided secretory production of cellulase in *T. reesei* into three main stages: Stage 1, transcription; Stage 2, secretion, including protein folding, transportation, post-translational modification, Endoplasmic reticulum-associated degradation (ERAD), etc., and Stage 3, cell metabolism such as amino acid precursor generation and intracellular redox homeostasis. Firstly, the main regulatory stage is at the transcriptional level, which involves the main activators Xyr1 [15], ACEII [16], and Hap2/3/5 [17], the repressors Cre1 and ACEI, and other newly identified ones [18–20]. Moreover, the secretion pathway, which contains many components such as chaperones, protein foldases, and SNARE proteins, is also important in controlling cellulase production. ERAD is one molecular mechanism that assists *T. reesei* to respond to secretion stress and is responsible for removal of misfolded proteins, thus serving as a quality-control system in protein secretion. Engineering protein folding and transportation can effectively promote secretive protein production in filamentous fungi such as *T. reesei* [21] and *Aspergillus awamori* [22]. Protein degradation and glycosylation also affect secretory protein production in the yeasts *S. cerevisiae* and *Pichia pastoris* [23, 24]. However, there have been very few studies previously on engineering protein degradation and glycosylation pathways to enhance cellulase production in *T. reesei*. Therefore, the related putatively functional genes were excellent models of this proof-of-concept analysis and, therefore, included for analysis. Furthermore, one frequently overlooked stage controlling cellulase production in *T. reesei* is the cell metabolism. Production of proteins results in a heavy metabolic burden to the cells, likely leading to a limitation in the supply of amino acids (the building blocks of proteins) and redox equivalents. While biosynthesis of amino acids is a complex metabolic process, both oxidative refolding of disulfide bonds and protein transport through the secretory machinery require energy. Additionally, controlling the redox state in the cytosol and ER is proved to be a prerequisite for efficient folding and secretion of proteins in *yeast* [25, 26]. Therefore, in this study, discovered or putatively functional genes within these three stages (transcription, secretion, and cell metabolism) were amplified and cloned into plasmids for overexpression or RNAi-mediated gene silencing. Pooled genes within each stage were simultaneously transformed into *T. reesei*, the fungus was iteratively evolved using this strategy, and the offspring strains were evaluated for their ability to produce cellulase.

## Results

### Designing an iterative semi-rational strategy for stepwise improvement of cellulase production in *T. reesei*.

**Fig. 1:**
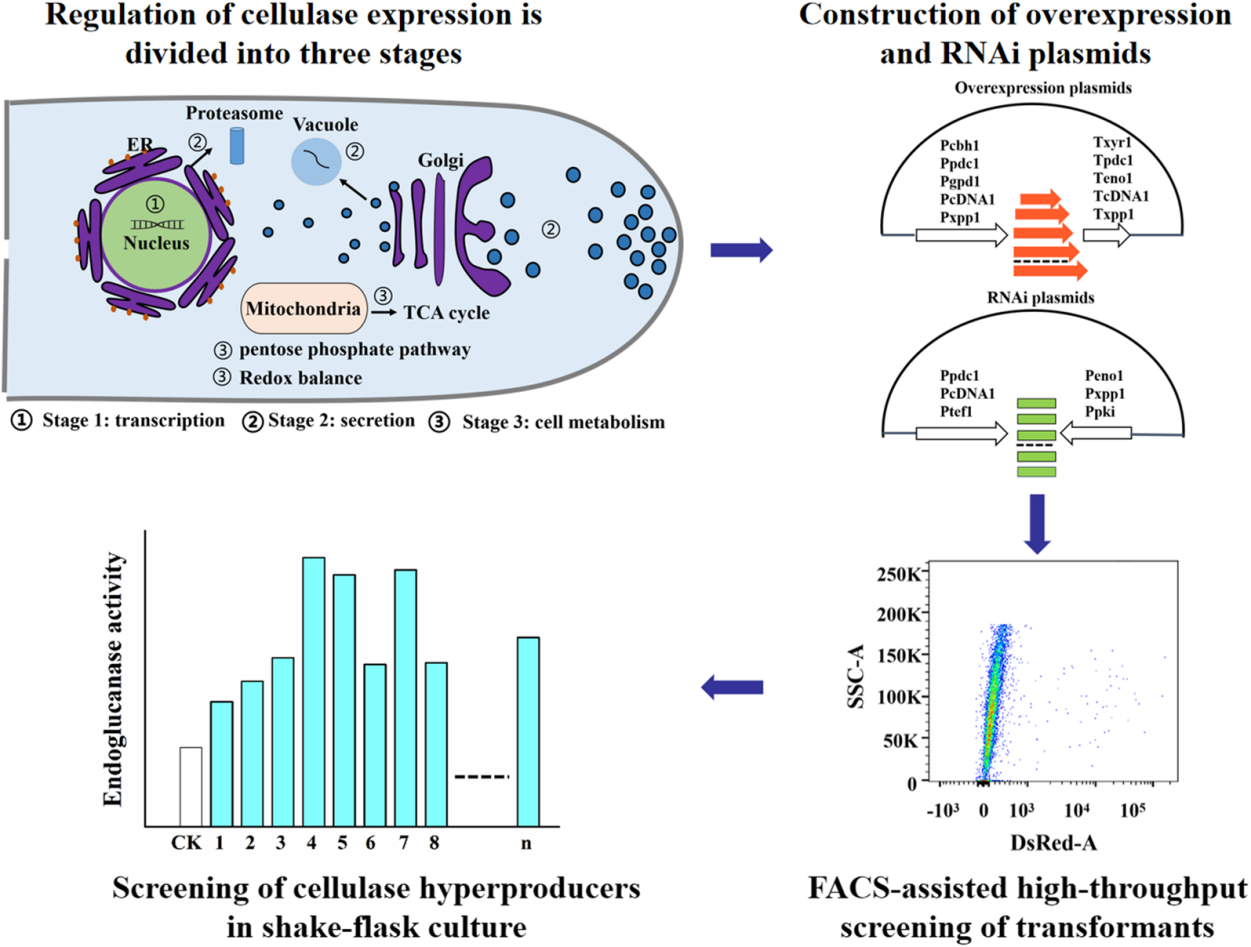
Schematic diagram showing iterative manipulation of multiple selected genes within the same stage at a time to improve cellulase production in *T. reesei*

The schematic diagram for iterative semi-rational manipulation of multiple selected genes at a time to improve cellulase production in *T. reesei* is shown in Fig. 1. Briefly, regulation of cellulase production was arbitrarily divided into three main stages. Experimentally verified or predicted activators were overexpressed, while the repressors were silenced for their expression. The plasmids bearing the genes in one same stage were pooled and co-transformed into a *T. reesei* strain (SUS4) with surface-displayed DsRed [27]. Transformant spores or protoplasts were screened for cellulase hyperproducers via fluorescence-assisted cell sorting (FACS, Fig. 1).

For manipulating the first stage which is widely accepted as the main stage of regulation for cellulase production in *T. reesei*, two well-studied transcription activators *xyr1* and *ace3*, as well as several other candidate regulators genes (*26163*, *66966*, *122523*, *80291*, *64608*, *123668*, *74765*, and *27600)* that might positively regulate cellulase production were selected as the targets of overexpression [19]. Transcription repressors including *cre1* and *ace1* were included for RNAi-mediated gene silencing. For the second stage, we integrated the data from other researchers’ lab [13] as well as from ours and selected homologous genes of *sed1*, *der1*, *ych1*, *ymr1*, *och1-2*, *pep4*, *doa10*, and *yps1* as candidate genes for gene silencing. For the third stage, the influence of metabolic flux towards generation of amino acid precursors and redox power on protein expression cannot be ignored in cellulase production. Therefore, we selected three types of genes as candidate genes, including (i) the key genes that contribute to generation of amino acid precursors in the TCA cycle (citrate synthase gene *80621* and isocitrate dehydrogenase gene *52055*); (ii) the genes for NADPH production in the pentose phosphate pathway (glucose-6-phosphate dehydrogenase *75769*, 6-phosphogluconolactonase *73903*, and 6-phosphogluconate dehydrogenase *72685*, for redox balance), and (iii) others such as glutathione reductase gene *53567*, for elimination free radicals and the *aox1* gene *57940*.

### Constructing plasmid pools to regulate cellulase production in *T. reesei*

All plasmids were constructed by using the Gibson assembly method in this study unless otherwise mentioned. To obtain the plasmids for overexpressing selected candidate genes, intermediate plasmids with six different sets of promoter/terminator were first constructed, which were Ppdc1/Tpdc1, Pgpd1/Teno1, PcDNA1/TcDNA1, Pxpp1/Txpp1, Ppki1/Tcbh1, and Pcbh1/Txyr1. The candidate genes that were thought to work positively were *xyr1*, *ace3*, *26163*, *66966*, *122523*, *80291*, *64608*, *123668*, *27600*, *74765*, *80621*, *53567*, *75769*, *72685*, *73903*, *52055*, and *57940* (Table 1). They were individually inserted into one of the plasmids. For gene silencing, three intermediate plasmids with two opposing strong constitutive promoters, i.e., pPpdc1-Peno1, pPcDNA1-Pxpp1, and pPtef1-Ppki, were constructed. The candidate genes were *ace1*, *cre1*, *sed1*, *der1*, *ych1*, *ymr1*, *och1-2*, *pep4*, *doa10* and *yps1* which were demonstrated or hypothesized to negatively affect cellulase secretory production (Table 1). They were inserted into the opposing promoters.

**Table 1:** The genes selected for use in this study

Stage	Gene	Verified or predicted functions	Genetic manipulation	Promoter	Terminator	Opposed promoter	References
1: transcription	*xyr1* (*122208*)	Transcription activator, verified	OE	*cbh1*	*xyr1*	N/A	[15]
	*ace3* (*77513*)	Transcription activator, verified	OE	*pdc1*	*pdc1*	N/A	[19]
	*26163*	Transcription activator, verified	OE	*gpd1*	*eno1*	N/A	[19]
	*66966*	Transcription activator, verified	OE	*gpd1*	*eno1*	N/A	[19]
	*122523*	Transcription activator, verified	OE	*gpd1*	*eno1*	N/A	[19]
	*80291*	Transcription activator, verified	OE	*cDNA1*	*cDNA1*	N/A	[19]
	*64608*	Transcription activator, verified	OE	*cDNA1*	*cDNA1*	N/A	[19]
	*123668*	Transcription activator, verified	OE	*xpp1*	*xpp1*	N/A	[19]
	*27600*	Transcription activator, predicted	OE	*xpp1*	*xpp1*	N/A	[45]
	*74765*	Transcription activator, verified	OE	*xpp1*	*xpp1*	N/A	[19]
	*cre1* (*120117*)	Transcription repressor, verified	RMGS	*cDNA1*	N/A	*xpp1*	[45]
	*ace1* (*75418*)	Transcription repressor, verified	RMGS	*cDNA1*	N/A	*xpp1*	[58]
2: secretion	*sed1* (*119975*)	Glycosylation, predicted	RMGS	*pdc1*	N/A	*eno1*	[13]
	*der1* (*124187*)	Protein degradation, verified	RMGS	*pdc1*	N/A	*eno1*	[38]
	*ych1* (*68274*)	Protein degradation, predicted	RMGS	*pdc1*	N/A	*eno1*	[13]
	*ymr1* (*119854*)	Protein degradation, predicted	RMGS	*tef1*	N/A	*pki*	[13]
	*och1-2* (*80340*)	Glycosylation, predicted	RMGS	*tef1*	N/A	*pki*	[13]
	*pep4* (*77579*)	Protein degradation, verified	RMGS	*tef1*	N/A	*pki*	[13]
	*doa10* (*123493*)	Protein degradation, verified	RMGS	*tef1*	N/A	*pki*	[59]
	*yps1* (*122076*)	Protein degradation, verified	RMGS	*cDNA1*	N/A	*xpp1*	[13]
3:cell metabolism	*80621*	Amino acid biogenesis, predicted	OE	*gpd1*	*eno1*	N/A	[60]
	*52055*	Amino acid biogenesis, predicted	OE	*pki*	*cbh2*	N/A	[61]
	*53567*	Free radical elimination, predicted	OE	*cDNA1*	*cDNA1*	N/A	[62]
	*zwf1* (*75769*)	NADP/NADPH balance, verified	OE	*cDNA1*	*cDNA1*	N/A	[37]
	*72685*	NADP/NADPH balance, verified	OE	*cDNA1*	*cDNA1*	N/A	[37]
	*73903*	NADP/NADPH balance, verified	OE	*pki*	*cbh2*	N/A	[26]
	*aox1* (*57940*)	NAD/NADH balance, verified	OE	*xpp1*	*xpp1*	N/A	[47]

*OE* overexpression, *RMGS* RNAi-mediated gene silencing, *N/A* not applicable

### Improving *T. reesei* cellulase production by manipulating the first regulatory stage

**Fig. 2:**
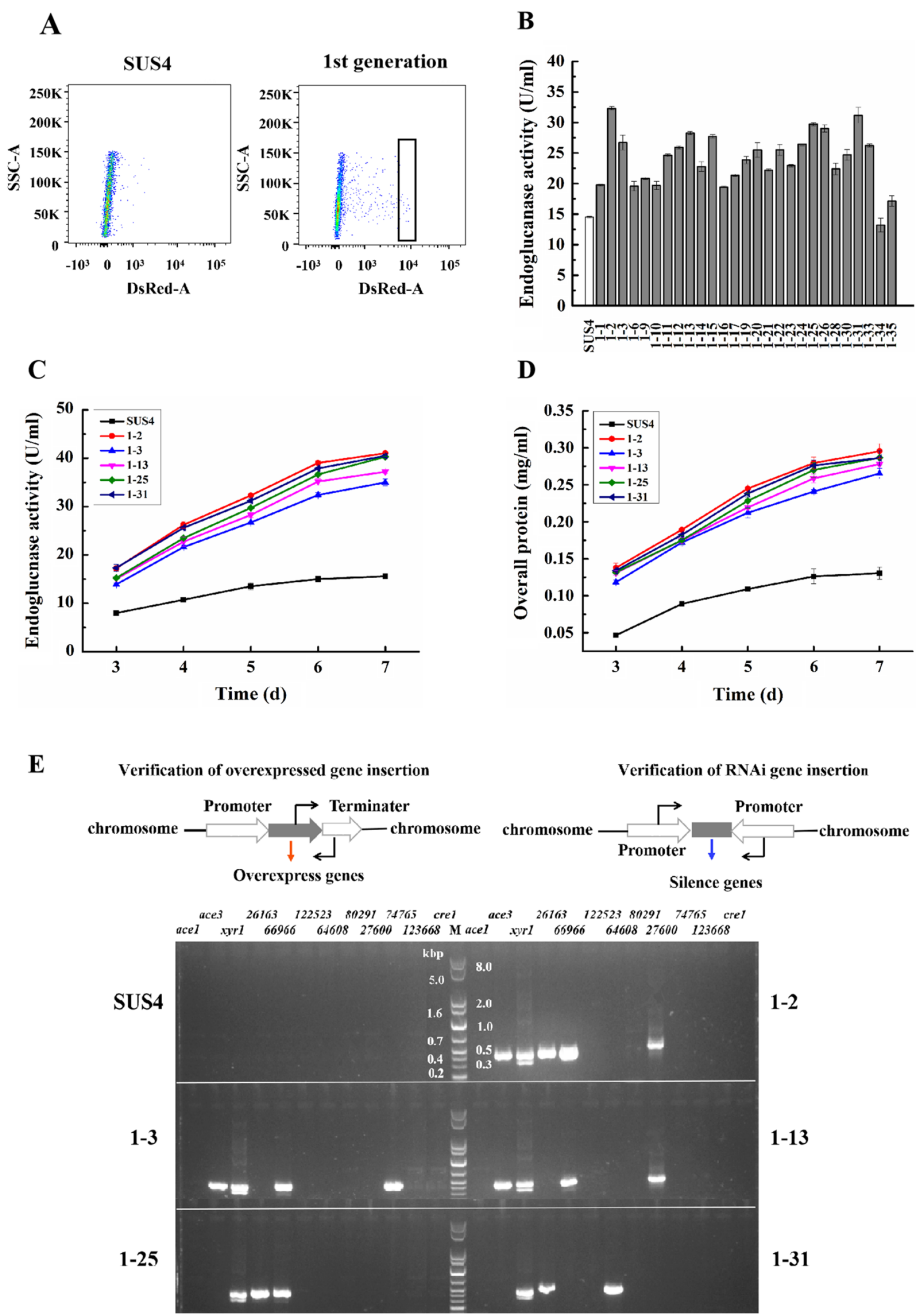
Manipulating Stage 1 to improve cellulase production. **A** Analysis of the transformant spores in FACS. The box indicated the spore sampling. **B** An initial screening of endoglucanase activity in the selected transformants on day 5 post-cellulase induction. **C** and **D** Production of endoglucanase (**C**) and extracellular protein concentration (**D**) of the transformants. **E** Determining integration of the transformed genes

In this study, the *T. reesei* strain SUS4 displaying the red fluorescence protein *Ds*Red on the cell surface was used as the host strain. In previous studies, we have shown that expression of the *DsRed* gene directed by the strong inducible *cbh1* promoter is positively related to cellulase production [27]. This allowed us to high-throughputly isolate cellulase hyperproducers from its transformants. While *xyr1* and *ace3* are well-characterized transcription activators, *cre1* and *ace1* are transcription repressors. Additionally, several putatively positively acting transcription factors (*26163*, *66966, 122523, 80291, 64608, 123668, 74765* and *27600*) were selected for testing in this study. In the first trial, the same amount of the plasmids for manipulating the first regulatory stage were mixed with a *pyr4-*expressing cassette [28] and used for SUS4 transformation. The transformants were inoculated on PDA plates for sporulation. The transformant spores were pooled and cultured with shaking for 13 h in liquid MM-lactose/sophorose medium. Flow cytometry analysis indicated that some of the transformants had remarkably higher red fluorescence signal than SUS4 (Fig. 2A). Twenty-seven transformants with highest red fluorescence were collected for sporulation. Germinated spores were cultured in the liquid cellulase-inducing medium for assay of cellulase activity. On day 5 post-cellulose induction, the cellulase activity as indicated by endoglucanase of most sorted mutants was higher than that of the parent strain in a preliminary screening (Fig. 2B). Five representative strains were chosen for further analysis of cellulase production and they were determined to indeed produce higher levels of cellulase (Fig. 2C) and extracellular proteins (Fig. 2D). On day 5 post-cellulose induction, compared with SUS4 (13.5 U/ml as the highest activity), the endoglucanase activity of the five strains increased to maxima of 26.7–32.3 U/ml, and the extracellular protein concentration reached to maxima of 0.21–0.25 mg/ml, which was approximately twice that of SUS4.

### Analyzing the integrated transcription regulatory genes in the representative transformants

The five representative strains (1–2, 1–3, 1–13, 1–25, and 1–31) and the parental strain SUS4 were used for extraction of genomic DNA and PCR amplification of the transformed DNA inserts. For the 12 selected genes, gene-specific primers were designed in positions located between the promoter and the gene or those between the gene and the terminator in the plasmids, with an expected size of amplification of ~ 500-bp (Fig. 2E). In the parental strain SUS4, there was no band corresponding to any of the 12 constructs. However, in all the transformants, there were either one or multiple DNA bands corresponding to the expected sizes of *ace3*, *xyr1*, *26163*, *66966*, *64608*, *27600*, and *74765* (Fig. 2E). For the representative transformant 1–2, 5 genes (*ace3*, *xyr1*, *26163*, *66966*, and *27600*) were introduced; for 1–3 and 1–13, two different sets of genes including *ace3*, *xyr1*, *66966*, and *74765* or *ace3*, *xyr1*, *66966*, and *27600* were inserted in the genome; for 1–25 and 1–31, two different sets each containing three genes (i.e., “*xyr1*, *26163*, and *66966*” and “*xyr1*, *26163*, and *64608*”) were introduced. Interestingly, all these transformants were introduced with the *xyr1* transcription activator gene. In addition, the relative transcript levels of the inserted genes in representative transformants were assayed. The transcript levels of these genes in SUS4 at 24 h post-induction were arbitrarily set as 1.0. The transcript abundance of almost all the inserted genes was significantly increased. The transcription of *xyr1*, *ace3*, *26163*, *66966*, and *27600* of 1–2 increased by 13.5 ± 1.1-, 2.9 ± 0.3-, 2.9 ± 0.2-, 10.4 ± 1.7-, and 3.2 ± 0.1-fold, respectively (Table 2). For some genes in specific transformants, there was no significant change, such as *66966* in 1–3, *26163* in 1–25, and *64608* in 1–31.

**Table 2:** The relative transcript abundance of transformed genes*

Stage	Gene	Parental strain	Representative transformants				
1: transcription		SUS4	1–2	1–3	1–13	1–25	1–31
	*ace3*	1.0	2.9 ± 0.3	2.2 ± 0.2	3.6 ± 0.2	N.D	N.D
	*xyr1*	1.0	13.5 ± 1.1	8.6 ± 0.6	7.6 ± 0.6	8.8 ± 1.1	9.5 ± 0.7
	*26163*	1.0	2.9 ± 0.2	N.D	N.D	1.1 ± 0.1	5.8 ± 0.6
	*66966*	1.0	10.4 ± 1.7	1.0 ± 0.2	17.0 ± 1.2	2.1 ± 0.3	N.D
	*27600*	1.0	3.2 ± 0.1	N.D	10.5 ± 0.3	N.D	N.D
	*74765*	1.0	N.D	8.3 ± 0.4	N.D	N.D	N.D
	*64608*	1.0	N.D	N.D	N.D	N.D	1.0 ± 0.1
2: secretion		1–2/*Δ*pyr4	2–3	2–4	2–13	2–18	2–29
	*sed1*	1.0	0.3 ± 0.0	1.4 ± 0.1	0.4 ± 0.1	0.6 ± 0.1	0.2 ± 0.1
	*der1*	1.0	1.5 ± 0.2	0.6 ± 0.1	0.8 ± 0.1	1.5 ± 0.2	0.9 ± 0.0
	*ych1*	1.0	1.1 ± 0.1	1.5 ± 0.2	1.5 ± 0.1	0.4 ± 0.1	0.9 ± 0.1
	*ymr1*	1.0	0.3 ± 0.1	0.3 ± 0.1	0.2 ± 0.0	0.5 ± 0.1	0.7 ± 0.2
	*och1-2*	1.0	1.1 ± 0.2	1.2 ± 0.1	0.4 ± 0.1	0.3 ± 0.0	0.2 ± 0.0
	*pep4*	1.0	0.2 ± 0.1	0.8 ± 0.1	0.8 ± 0.2	0.8 ± 0.1	0.7 ± 0.1
	*doa10*	1.0	0.6 ± 0.1	0.8 ± 0.1	1.6 ± 0.3	0.9 ± 0.1	1.2 ± 0.2
	*yps1*	1.0	0.3 ± 0.1	0.8 ± 0.1	0.7 ± 0.1	1.7 ± 0.2	1.3 ± 0.1
3: cell metabolism		2–3/*Δ*pyr4	3–2	3–4	3–8	3–18	3–25
	*80621*	1.0	1.1 ± 0.2	2.3 ± 0.2	1.1 ± 0.1	1.2 ± 0.1	0.9 ± 0.2
	*52055*	1.0	2.1 ± 0.3	0.9 ± 0.1	1.3 ± 0.2	1.1 ± 0.1	1.2 ± 0.0
	*53567*	1.0	2.0 ± 0.2	1.3 ± 0.1	2.9 ± 0.3	2.3 ± 0.1	1.2 ± 0.1
	*75769*	1.0	2.3 ± 02	1.1 ± 0.1	5.0 ± 0.3	2.4 ± 0.2	1.3 ± 0.1
	*72685*	1.0	8.6 ± 0.2	3.0 ± 0.2	4.0 ± 0.2	4.1 ± 0.4	6.5 ± 0.2
	*73903*	1.0	2.4 ± 0.3	1.1 ± 0.0	2.7 ± 0.1	2.6 ± 0.2	1.6 ± 0.1
	*57940*	1.0	2.9 ± 0.1	0.7 ± 0.2	0.5 ± 0.1	0.8 ± 0.1	0.9 ± 0.0

^*^All experiments were performed in three independent replicates

*N.D.* not determined

### Manipulating expression of selected genes in Stage 2

The 1–2 representative transformant was selected to test if simultaneous manipulating expression of multiple genes involved in the secretion pathway would similarly improve cellulase production. The two direct repeats flanking *pyr4* enabled us to rapidly loop out the selection marker gene of this strain by placing the spores of the strain on minimal medium plates.

**Fig. 3:**
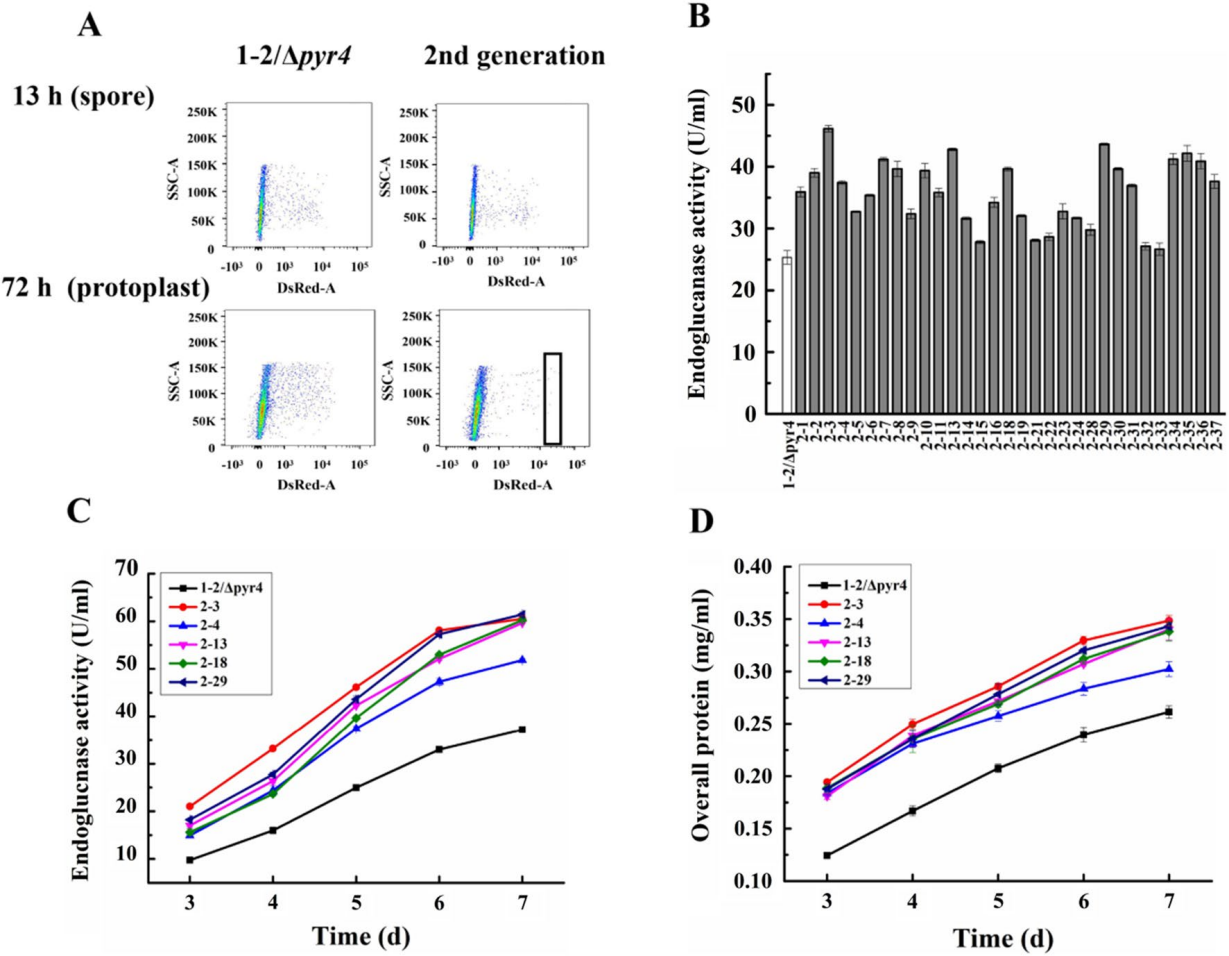
Manipulating Stage 2 to improve cellulase production. **A** FACS analysis of the transformant spores at 13 h post-induction or protoplasts prepared from the transformant mycelia at 72 h post-induction. The box indicated the protoplast sampling. **B** An initial screening of endoglucanase activity in the selected transformants on day 5. **C** and **D**. Production of endoglucanase (**C**) and extracellular protein concentration (**D**) of the transformants

Previously we demonstrated that overexpressing chaperone or SNARE genes in the secretion pathway can significantly improve cellulase [27] as well as heterologous protein [21] production. Herein, for regulation of Stage 2, we instead silenced genes (*sed1*, *der1*, *ych1*, *ymr1*, *och1-2*, *pep4*, *doa10* and *yps1*) associated with the glycosylation and ERAD process. These genes included those encoding the intracellular proteases that are proposed to be involved in quality control of folding of secretory proteins. The uridine auxotroph mutant of strain 1–2 (named 1–2/*Δpyr4*) with nearly identical cellulase-producing ability to that of strain 1–2 was transformed with a pool of plasmids silencing these candidate genes in regulatory Stage 2. Unfortunately, in the initial trials when using germinated spores for FACS analysis, the transformants displayed nearly identical red fluorescence signal to that of 1–2/*Δpyr4* (Fig. 3A).

Since most of the selected genes were involved in protein secretion such as glycosylation and intracellular protein degradation, we hypothesized that the induction time as short as 13 h for the germinated spores may not be long enough for differentiating the red fluorescence. Therefore, the mixed spores were cultured for an extended period of 72 h when protein began to be largely secreted and the mycelia were enzymatically treated for protoplast releasing. With this modification, part of the transformed protoplasts displayed higher red fluorescence signal than 1–2/*Δpyr4* in FACS analysis (Fig. 3A). The survival rate of individual protoplasts ranged from 30 to 40%. On day 5 post-cellulose induction in shake-flask cultivation, all sorted transformants showed higher endoglucanase activity than 1–2/*Δpyr4* (Fig. 3B). Five representative strains displayed higher ability to produce cellulase (Fig. 3C) and extracellular proteins (Fig. 3D) in this flask cultivation. Compared with the parent strain 1–2/*Δpyr4* (26.0 U/ml), the endoglucanase activity of the five representative strains was increased to 37.4 U/ml (2–4) ~ 46.2 U/ml (2–3) on day 5 post-induction (Fig. 2C). Not surprisingly, the overall extracellular protein of these transformants reached maximal concentration of 0.26 ~ 0.30 mg/ml, higher than 0.21 mg/ml for 1–2/*Δ*pyr4.

From the RT-qPCR analysis, decreased expression of the functional genes was observed in each of the representative transformant (Table 2). For example, the relative transcript levels of *sed1*, *ymr1*, *pep4*, *doa10*, and *yps1* in strain 2–3 were much lower than those in 1–2/*Δ*pyr4, decreasing to 0.3 ± 0.0-, 0.3 ± 0.1-, 0.2 ± 0.1-, 0.6 ± 0.1-, and 0.3 ± 0.1-fold, respectively. Repressed expression of *ymr1*, but not the others, was observed in all five offspring strains.

### Manipulating expression of selected genes in Stage 3 for further improved cellulase production

**Fig. 4:**
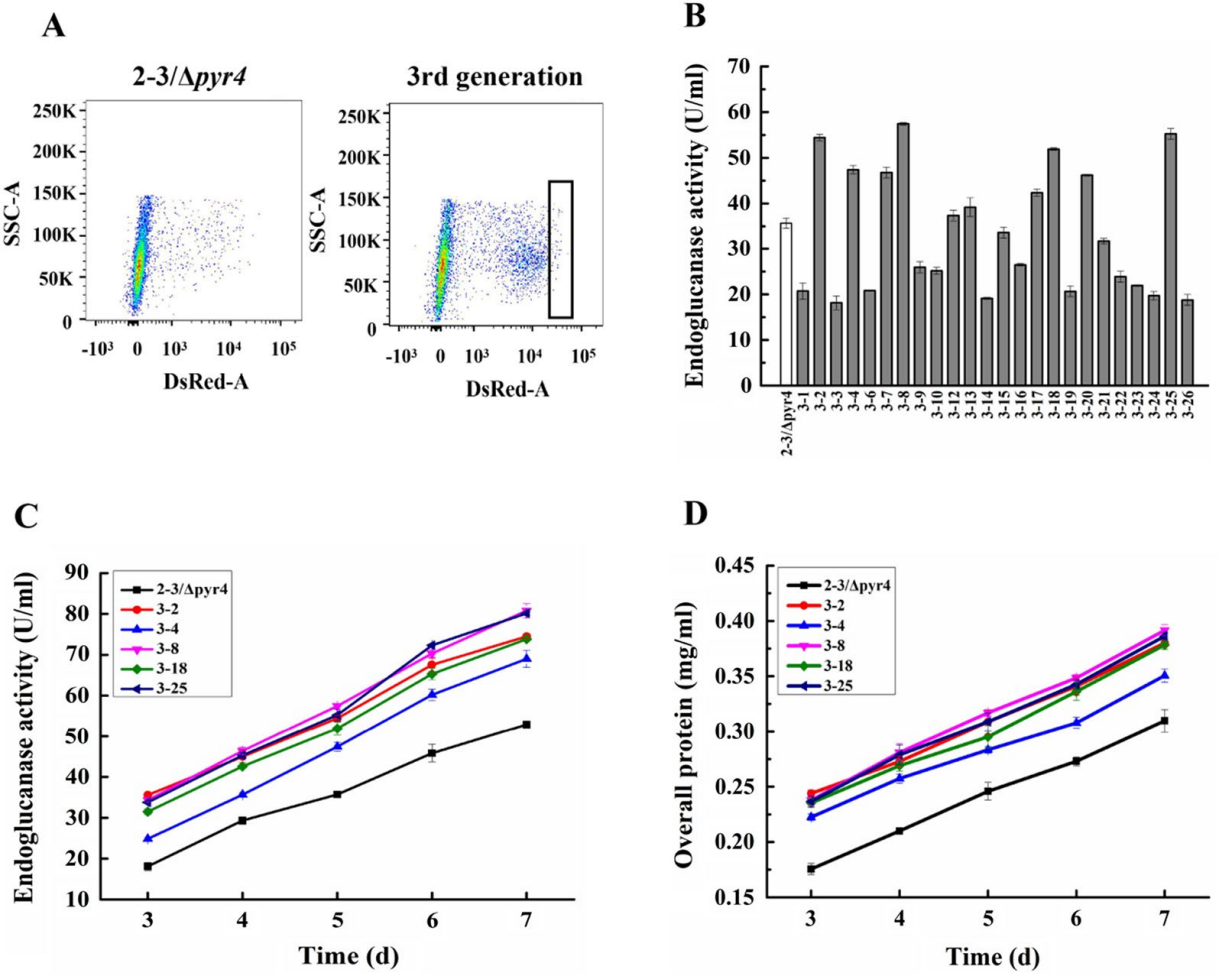
Manipulating Stage 3 to improve cellulase production. **A** FACS analysis of the protoplasts prepared from the transformant mycelia at 72 h post-cellulase induction. The box indicated the protoplast sampling. **B** An initial screening of endoglucanase activity in the selected transformants on day 5. **C** and **D** Production of endoglucanase (**C**) and extracellular protein concentration (**D**) of the transformants

Strain 2–3 was selected for further engineering. The *pyr4* auxotrophic mutant of strain 2–3 was prepared and introduced with plasmids for overexpressing the seven genes (*80621*-citrate synthase, *52055*-isocitrate dehydrogenase, *53567*-glutathione reductase, *75769-*glucose-6-phosphate dehydrogenase, *72685–*6-phosphogluconate dehydrogenase, *73903*–6-gluconolactonase, and *57940*-alternative oxidase) targeting stage 3 for regulation (Table 1). FACS analysis of the protoplasts, rather than the germinated spores, indicated that there were transformants with stronger red fluorescence than the direct parental strain 2–3/*Δpyr4* (Fig. 4A). Part of the sorted transformants showed higher cellulase activity than 2–3/*Δpyr4* (Fig. 4B). The cellulase activities of five selected transformants topped at 47.4 (for 3–4)-57.5 (for 3–8) U/ml, significantly higher than that of strain 2–3/*Δpyr4* (35.7 U/ml) on day 5 post-cellulose induction (Fig. 4C). The extracellular protein concentrations of the five representative strains ranged in 0.28 ~ 0.33 mg/ml, also higher than that of the parent strain 2–3/*Δpyr4* (0.25 mg/ml) (Fig. 4D).

In the RT-qPCR analysis, the relative transcript levels of genes *53567*, *75769*, *72685*, and *73903* in the 3–8 transformant were 2.9 ± 0.3-, 5.0 ± 0.3-, 4.0 ± 0.2-, and 2.7 ± 0.1-fold that of strain 2–3/*Δpyr4* (Table 2). Interestingly, the pentose phosphate pathway, particularly the gene *72685* appeared to be enriched in the selected transformants.

### Expression of cellulase genes in WT and the 1st, 2nd, and 3rd generation transformants

**Fig. 5:**
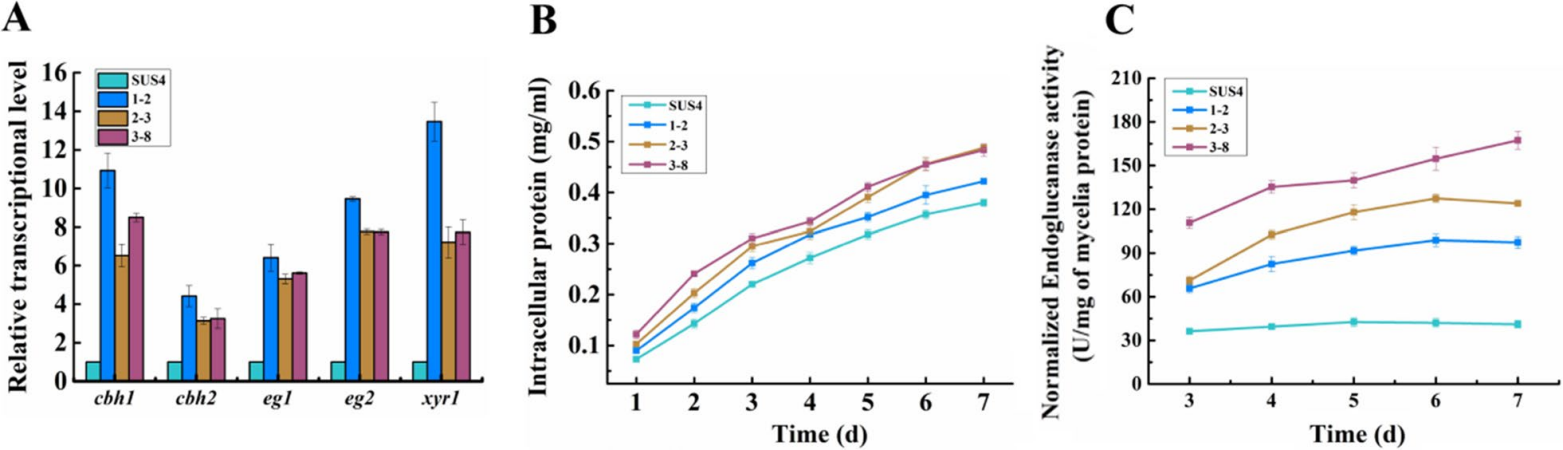
Comparison of cellulase production in WT and three representative strains. **A** Relative transcript levels of the main cellulase genes *cbh1*, *cbh2*, *egl1*, *egl2* and the master transcription activator *xyr1*, which were determined at 24 h post-cellulase induction. **B** Intracellular protein concentrations (as a reflection of the mycelial biomass) of the transformants. **C** Normalized endoglucanase activity against fungal biomass

Among the three selected stage transformants, 1–2, 2–3, and 3–8 transformants with comparably higher endoglucanase activity were selected as representative strains of the 1st, 2nd and 3rd round manipulation of transformants, respectively. The increase in cellulase production could be visualized on an SDS-PAGE gel of 1–2, 2–3, and 3–8 transformants on day 5 post-cellulose induction (Additional file 1: Figure S2). The relative transcript level of *cbh1*, *cbh2*, *eg1*, *eg2*, and *xyr1* of WT cultured for 24 h in the cellulase-inducing medium was arbitrarily set as 1.0. Clearly, the relative transcript levels of *cbh1*, *cbh2*, *eg1*, *eg2*, and *xyr1* of 1–2 were much higher in the genetically engineered strains than those of WT, increasing by 10.9-fold, 4.4-fold, 6.4-fold, 9.5-fold, and 13.5-fold, respectively (Fig. 5A).

We also noticed that the intracellular protein concentrations (as a reflection of the mycelia biomass) of the engineered strains were slightly higher than that of the WT strain SUS4 (Fig. 5B). For example, on day 5 post-cellulose induction, the intracellular proteins of WT, 1–2, 2–3, and 3–8 were 0.32, 0.35, 0.40, and 0.41 mg/ml, respectively. However, the larger amount of biomass cannot account for all the increments in cellulase-producing ability. The normalized endoglucanase activity against mycelial biomass in 1–2, 2–3, and 3–8 all increased when comparing to their parental strains (Fig. 5C). For example, on day 5 post-cellulose induction, the normalized endoglucanase activity of SUS4, 1–2, 2–3, and 3–8 were 42.6, 91.8, 117.9, and 139.8 U/mg, respectively. Therefore, the improvement of cellulase-producing ability of the engineered strains was a combinatorial effect of increments in the specific cellulase-production rate and growth rate.

## Discussion

Strain improvement is critical to modern biotechnology and multiple rounds of engineering are often demanded for development of a highly efficient protein production strain. In this study, we used *T. reesei* as a model filamentous fungus and demonstrated as a proof-of-concept that genes within an arbitrarily defined regulatory stage could be pooled to stimulate secretory protein production, and moreover, this method could be iteratively used for evolving an even higher ability of protein production. This strategy is semi-rational, which essentially does not rely on established understanding of the deliberate regulatory mechanisms of secretory protein expression. Indeed, as demonstrated in Stage 2 and 3, when the regulation is not clear in emerging non-canonical filamentous fungal hosts such as in *H. insolens* and *M. thermophila*, one can learn from the other organisms whose mechanisms have been elucidated and accordingly use the homologous genes for evolution. In our study, RNAi was used to silence expression of repressors. RNAi was well-known to lead to varying levels of targeted gene suppression [29], thereby further extending the diversity in the transcript levels of selected genes. The RNAi machinery components appear to be universal in many filamentous fungi [30] and thus can be similarly employed in the semi-rational evolution.

Manipulation of the transcription stage led to the most significant improvement. The cellulase activity increased to up to 2.4-fold and the protein concentration increased to up to 2.3-fold. This is in accordance with the notion that cellulase induction in *T. reesei* is mainly regulated at the transcription level [31]. The predominant presence of the master transcription activator *xyr1* transcript indicated that overexpression of *xyr1* played a major role in the cellulase hyperproducer strains. However, when *xyr1* was individually transformed in SUS4, the cellulase activity and extracellular protein concentration of three representative transformants increased by 1.6 ~ 1.7-fold and 1.5-fold, respectively (Additional file 1: Figure S1A, B), which were much smaller than those obtained by co-transformation of *xyr1* and other selected genes. The normalized cellulase activity against mycelial biomass was also increased in the transformants (Additional file 1: Fig. S1C). In the three *xyr1*-overexpressing transformants, the *xyr1* transcript level increased to a level (8.7 ~ 10.6-fold) similar to those strains obtained in manipulating Stage 1 (Additional file 1: Fig. S1D). The results suggested that combining specific groups of genes might be superior to manipulating just one master regulator. However, we cannot exclude the possibility that in the co-transformed genes, there could be one or more that either did not impact or even negatively affected cellulase expression.

Unexpectedly, although the well-characterized transcription activator gene *ace3* was detected in three of the transformants, *66966* was discovered in four of them and overexpressed in three. This strongly suggested that gene *66966* might act to stimulate cellulase transcription in *T. reesei*. The gene *66966* was selected from the transcriptomics data because the cellulase activity of a strain overexpressing this gene was more than 1.5 times that of the parent strain and predicted to encode a conserved WD40 repeat-containing protein with unknown functions [19]. WD40 proteins play important roles in a variety of cellular functions including signal transduction, chromatin remodeling, and transcriptional regulation [32]. In addition, although overexpression of the gene *26163* (the homologue of *clr-2*) did not lead to significant improvement in cellulase production in *T. reesei* QM9414 [19], its transcript level significantly increased in 1–2 and 1–31. In the genome, the gene is located close to a sugar transporter, which is considered to be a lactose permease essential for induction of *cbh1* and *cbh2* [33]. However, the contribution of this gene to improved cellulase activity, especially in a strain with a genetic background different from QM9414, remains to be elucidated.

We further discovered that in all stages (Stage 1–3) a certain selected gene(s) were prevalent in the selected representative transformants. For example, *xyr1* appeared in all five Stage 1 transformants, *ymr1* was observed in four of the five Stage 2 transformants, and gene *72685* was present in all five Stage 3 transformants. Since *xyr1* is a main transcriptional activator that regulates cellulase production, it is not surprising that *xyr1* appeared in all five Stage 1 transformants. *ymr1* (yeast myotubularin-related phosphatase) is a myotubularin family member and has a direct role in regulation of phosphatidylinositol 3-phosphate-dependent signaling pathways [34]. It has been reported that phosphatidylinositol 3-phosphate serves as a key regulator of vesicular trafficking within the endosomal system [35]. Therefore, interfering with *ymr1* expression may affect protein translocation from late Golgi apparatus to the vacuole, thus affecting cellulase secretion. The gene *72685* (6-phosphogluconate dehydrogenase) is a key regulator in the pentose phosphate pathway, a catabolic pathway known to produce NADPH. NADPH is known to be an important cofactor ensuring intracellular redox balance [36]. The gene *72685* was present in all five Stage 3 transformants, suggesting that alteration of the pentose phosphate pathway could change the metabolic flux and further fine-tune the intracellular redox balance. These results were consistent with the reports that overexpressing *gndA* gene (6-phosphogluconate dehydrogenase) in *A. niger* can increase the yield of glucoamylase [37]. Therefore, it would be particularly intriguing to investigate whether and how these genes can stimulate cellulase gene expression and whether they can interact, either physically or genetically, with other known regulators responsible for cellulase production.

The obtaining of improved transformants in manipulating Stage 2 suggested that the poorly understood ERAD might not be perfect. As a result, attenuating this pathway appeared to counteract this quality-control mechanism and in future, may be used in combination with overexpressing the ER-resident chaperones for enhancing secretory protein production [21]. Carvalho reported that, in *A. niger*, disruption of the ERAD pathway genes increased the intracellular concentration of the glucoamylase–glucuronidase (GlaGus) fusion reporter protein. However, the extracellular GlaGus appeared not to be increased [38]. Two reasons might account for the observed inability to improve protein secretion in *A. niger*. First, secreted GlaGus might be vulnerable to the *A. niger* extracellular proteases. Second, GlaGus in *A. niger* and cellulase in *T. reesei* likely require different transporting component proteins in the secretion pathway.

The finding that manipulating gene expression in Stage 3 could stimulate cellulase gene expression is encouraging. Note that, based on the same hypothesis as that for Stage 2, we utilized protoplasts instead of spores to screen for the cellulase hyperproducers. Improvement of cellulase-producing ability from Stage 2 to Stage 3 indicated that a fine-tuning of the interior cellular metabolism and redox state is required for efficient cellulase production. Since it had been proved that multiple genes could be integrated in the chromosome in Stage 1, investigation by PCR of the selected genes in Stage 2 and 3 was not carried out further. However, the success in stimulating more cellulase production may prompt more mechanistic investigations to be placed in how cellular metabolism can influence cellulase production in *T. reesei*. The transcription of genes involved in the pentose phosphate pathway (e.g., gene *72685*) was significantly enhanced in selected transformants, likely reflecting the important influence of NADP/NADPH balance on protein production. Indeed, several studies have already shown that fine-tuning the metabolic balance through change of the pentose phosphate pathway can improve heterologous protein expression, such as in *Pichia pastoris* [39] and *A. niger* [40].

Unexpectedly, the relative transcript levels of main cellulase (*cbh1*, *cbh2*, *eg1*, and *eg2*) and *xyr1* genes of 2–3 and 3–8 were lower than (but still comparable to) those of 1–2. This could be due to other unidentified mutations in the genome when trying to obtaining the *pyr4-* recipient strains. However, the possibility that altered gene expression at Stage 2 and 3 may affect cellulase production cannot be excluded. In manipulating functional genes in Stage 2, while more proteins might be retained in the endoplasmic reticulum and translocated to the Golgi apparatus, this led to an interference with the secretory pathway. Impairment of the pathway is known to be able to decrease expression of major cellulase genes through a mechanism known as repression under secretion stress (RESS) [41]. In addition, change of cell metabolism and redox state can also affect transcription of secretory protein. For example, in rat, repressing the function of isocitrate dehydrogenase-2 (IDH2, catalyzing reductive carboxylation of 2-ketoglutarate to isocitrate) lowered the NADPH level and inhibited insulin secretion [42, 43]. Such information, together with the impairment in cellulase production in knocking out *pyr4*, might account for the observation that transcription of some cellulase genes were less abundantly transcribed in the Stage 2 and 3 stains.

In modern strain engineering, robot-based automation such as biofoundry appears to be more and more involved. Due to its high efficiency, this technology deals often with strains from pooled genes in metabolic pathways (such as mutant libraries from directed evolution) transformation with highly diversified background [44]. Therefore, the success in *T. reesei* suggests that the semi-rational strategy may, as in unicellular microbes, also be used in filamentous fungi in robot-based high-throughput strain improvement. Importantly, the current study clearly pinpoints the possibility of using this method in emerging filamentous fungi hosts with little knowledge regarding regulation of protein secretion.

## Conclusions

In this study, we arbitrarily divided cellulase production in *T. reesei* in three main stages (transcription, secretion, and cell metabolism). Functionally validated or predicted functional genes potentially operating at the same stage were selected and co-transformed into the recipient *T. reesei* strains. We could demonstrate as a proof-of-concept that genes within an arbitrarily defined regulatory stage could be pooled to stimulate secretory cellulase production, and moreover, this method could be iteratively used. This strategy could also be used in filamentous fungi with poor knowledge about expression of secretory proteins.

## Methods

### Strains and plasmids

The *Escherichia coli* Fast-T1 strain from Vazyme (Nanjing, China) was used throughout this study for plasmid construction and propagation. *E. coli* was cultured in Luria–Bertani (LB) medium supplemented with an appropriate antibiotic if needed. The mutant strain *T. reesei* SUS4 expressing *Ds*Red on its cell surface [27] is a uridine auxotroph of SUS1 and was used as the recipient strain in this study. SUS1 is a mutant of QM9414 developed in our lab after multiple rounds of mutagenesis with an enhanced cellulase-producing ability and lower viscosity [28]. All *T. reesei* strains were maintained on potato dextrose agar (PDA) at 28 °C for sporulation. For enzyme production, the *T. reesei* strains were grown in a minimal medium (MM) containing (NH_4_)_2_SO_4_, 5.0 g/L; KH_2_PO_4_, 15 g/L; MgSO_4_, 0.6 g/L; CaCl_2_, 0.6 g/L; FeSO_4_·7H_2_O, 0.005 g/L; MnSO_4_·H_2_O, 0.0016 g/L; ZnSO_4_·7H_2_O, 0.0014 g/L; CoCl_2_, 0.002 g/L. MM was supplemented with glucose (2%) for mycelial growth or Avicel crystalline cellulose (2%) for cellulase induction.

### Selection of functional genes for simultaneous transformation

Expression of cellulase in *T. reesei* is affected by multiple regulatory stages. Based on literatures and publicly available transcriptomic data, the following candidate genes involved in transcription, secretion, and metabolism were selected. The well-characterized transcription activators *xyr1*, *ace3*, and several putative transcription factors including gene *26163*, *66966, 122523, 80291, 64608, 123668, 74765* [19]*,* the and *27600* [45] that may act positively were selected for overexpression. In addition, the known transcription repressors *cre1* and *ace1* were chosen for RNAi-mediated gene silencing. Several genes (*sed1*, *der1*, *ych1*, *ymr1*, *och1-2*, *pep4*, *doa10* and *yps1*) involved in protein secretion in *S. cerevisiae*. These genes mainly involved in the ERAD process were selected for RNAi-mediated gene silencing. The cellular metabolism is regarded to have strong impact on synthesis of secreted proteins. Therefore, genes including *80621* (citrate synthase gene), *52055* (isocitrate dehydrogenase gene), *53567* (glutathione reductase gene), *75,769* (glucose-6-phosphate dehydrogenase gene, *zwf1*)[37], *72685* (6-phosphogluconate dehydrogenase gene) [37], *73903* (6-gluconolactonase gene) [46], and *57940* (alternative oxidase gene, *aox1*) [47] involved in tricarboxylic acid cycle, redox process or pentose phosphate pathway were selected as candidate regulators (Table 1). These genes were overexpressed in *T. reesei*.

### Plasmid construction

We used the Gibson assembly [48], a method that utilizes the highly efficient in vitro homologous recombination machinery, to construct the plasmids in this study. To construct the plasmids overexpressing candidate genes, we first selected five strong promoters (*cbh1* [49], *pdc1* [50], *gpd1*[51], *cDNA1* [52], *xpp1* [53]) and terminators (*xyr1*, *pdc1*, *eno1*, *cDNA1*, and *xpp1*) to construct five intermediate plasmids which contained a promoter and a corresponding terminator. These promoters and terminators were amplified from the genomic DNA of *T. reesei*, and the ampicillin resistance gene and *E. coil* replication origin were amplified from pCbh1pWT-DsRed-TEL [54] (primers listed in Additional file 1: Table S1). Restriction enzymes *Sal*I, *Sna*BI, *Pac*I, and *Nde*I were added between the promoter and terminator for ease of inserting candidate genes. Then the four amplified DNA fragments were mixed with 2 × ClonExpress Mix (ClonExpress Ultra One Step Cloning Kit, Vazyme, Nanjing, China), incubated at 50 ºC for 15 min, and transformed into the *E. coli* competent cell Fast-T1. The resultant intermediate plasmids pPcbh1-Tcbh1, pPpdc1-Tpdc1, pPgpd1-Teno1, pPcDNA1-TcDNA1, pPXpp1-TXpp1, and pPpki-Tcbh2 were digested with two of the four restriction enzymes, respectively. The experimentally verified or putatively positively acting regulator genes (*xyr1*, *ace3, 26163, 66966, 122523, 80291, 64608, 123668, 27600, 74765, 80621, 53567, 75769, 72685, 73903, 52055,* and *57940*) were amplified from the genomic DNA of *T. reesei* and inserted using Gibson assembly into the intermediate plasmids (Table 1).

To construct the plasmids for RNAi-mediated gene silencing of the repressors, we used the head-to-head dual promoters design [55]. Three intermediate plasmids (pPpdc1-Peno1, pPcDNA1-PXpp1, and pPtef1-Ppki) were constructed by Gibson assembly method, each of which contained two head-to-head dual promoters. All these promoters were amplified from the genomic DNA of *T. reesei*, while the ampicillin resistance gene and *E. coil* replication origin were amplified from pCbh1pWT-DsRed-TEL (primers listed in Additional file 1: Table S1). Similarly, restriction enzymes *Sal*I, *Sna*BI, *Pac*I, and *Nde*I were added between the two promoters for ease of inserting the candidate genes. The constructed intermediate plasmids were restriction digested with two of the four enzymes. The verified or putatively negative regulators (*ace1*, *cre1*, *sed1*, *der1*, *ych1*, *ymr1*, *och1-2*, *pep4*, *doa10*, and *yps1*) were amplified from the genomic DNA of *T. reesei* and individually inserted into one of the intermediate plasmids to obtain the RNAi plasmids (Table 1). The direct repeats of ampicillin resistance genes containing the *pyr4* selection marker gene used for complementation of the uridine auxotrophy were amplified from the pAPA plasmid [28] with the primer pairs (listed in Additional file 1: Table S1).

### Transformation of *T. reesei*

The plasmids encoding the selected genes were introduced into *T. reesei* by following a modified method of polyethylene glycol (PEG)-mediated protoplast transformation [56]. Briefly, *T. reesei* was grown in MM-glucose (2%) at 28 °C for 20 h. Mycelia were collected and incubated with 10 mg/mL of Lysing Enzymes (L1412, Sigma-Aldrich, St. Louis, MO) plus 1 mg/mL of cellulase (ONOZUKAR-10) at 30 °C until large amounts of protoplasts were released. Two μg each of selected plasmids and the APA fragment (half amount of total plasmids added) were used to co-transform the SUS4 protoplasts. The transformants were selected on MM-glucose agar plates for 5 d. All transformant colonies were washed with sterile water and the mixed transformants were transferred to the potato dextrose agar (PDA) for re-sporulation. Spores were collected from the mixed transformants and used for FACS.

### FACS

For flow cytometry-assisted cell sorting of spores, fresh spores were transferred into liquid MM-lactose/sophorose (2% for lactose and 0.003% for sophorose, w/v) and shaken at 160 rpm for 13 h. Germinated spore suspensions were filtered through a 200-mesh sifter to remove long hyphae and chunks. High-speed sorting was performed in a FACS Aria sorter at a rate of 5,000 events per sec, 30 psi with an 85 m nozzle. Single cells with the brightest DsRed fluorescence signal (top 0.1%) were sorted into individual wells of a 6-well plate and cultured at 28 ºC for sporulation.

For FACS analysis of protoplasts, fresh spores of the transformants were cultured in liquid MM-2% glucose for 24 h and 1/10 (v/v) of the germinated spores were transferred to liquid MM-2% Avicel and shaken at 160 rpm for 72 h. Then, the hyphae were collected for preparing protoplasts. Protoplasts were prepared using the method essentially the same as above described and passed through the FACS instrument. Protoplasts with the highest fluorescence signal were directly sorted into individual wells of a 96-well plate containing 1 M sorbitol (to maintain the osmotic pressure) and MM with 2% glucose as the carbon source. The protoplasts were incubated for 96 h at 28 ºC with gentle shaking for regeneration of the cell wall and propagation. Then, 10 μl of the cell suspension from each of the 96-well plate were transferred on PDA plate and cultured at 28 ºC for sporulation.

### Induction of cellulase production

For induction of cellulase gene expression in *T. reesei*, 10^7^ fresh spores were inoculated into 50 ml of liquid MM supplemented with 2% glucose and shaken at 28 ºC for 36 h. The mycelia were filtered by passing the culture through a 200-mesh sifter and washed twice with MM to remove residual glucose. One gram of the mycelia was added to 100 ml of MM-Avicel (2%) for cellulase induction. From day 2 to 7 post-Avicel cellulose inoculation, 2 ml of the culture were periodically taken out for assay of the cellulase activities and protein concentrations.

### Assay of cellulase activity and protein concentration

Since SUS4 is a strain with *eg2*-overexpression [27], the endoglucanase activity was used as an indicator of cellulase activity. For assay of endoglucanase, sodium carboxymethyl cellulose (CMC-Na, from Sigma-Aldrich) was used as the substrate. CMC-Na was dissolved in a McIlvaine buffer (200 mM, pH 5.0). The reactions contained 900 μl of 1.5% (w/v) CMC-Na and 100 μl of appropriately diluted enzymes and they were incubated at 50 °C for 30 min. Then 1.5 ml of the DNS (3,5-dinitro-salicylic acid) reagent was added to the mixture and boiled for 5 min to terminate the reaction. One unit of endoglucanase activity was defined as the amount of enzyme that released 1 μmol of reducing sugar per minute under the assay conditions. The extracellular protein concentration of the fermentation supernatants was determined using BCA-200 Protein Assay Kit (Pierce, Rockford, IL). The fermentation broth for wild-type and transformants were also analyzed by sodium dodecyl sulfate–polyacrylamide gel electrophoresis (SDS-PAGE). For assay of mycelial biomass, we used mycelial proteins as representatives of the fungal biomass to avoid the interference of insoluble cellulose Avicel. The mycelia of the strains were collected in different time periods from days 1 to 7, and the method described by Jayaraman [57] was adopted to determine the mycelial proteins.

### Isolation of homokaryons and knockout of the *pyr4* gene

Selected *T. reesei* transformants were used for isolation of homokaryons by first sporulation on PDA plates. Then, 200 μl of each of spore suspensions of the transformants at dilution rates of 10^–1^, 10^–2^, 10^–3^, 10^–4^, 10^–5^, and 10^–6^ were spread on the MM-glucose (2%) agar plates containing 0.1% Triton X-100 and cultured for 4–5 d. For knocking out the *pyr4* gene in the transformants, 200 μl each of spore suspensions of the transformants at dilution rates of 10^–1^, 10^–2^, 10^–3^, 10^–4^, and 10^–5^ were spread on MM-glucose (2%) agar plates containing 3 mg/ml of 5-fluoroorotic acid (Biotopped, Beijing, China) and 10 mM uridine (Sigma) and cultured for 7 d. The transformants with *pyr4* gene knocked out were tested for cellulase activity, and those with a similar cellulase-producing ability to that of the original strain were selected for further study.

### Determining insertion of selected genes in the transcription stage in the transformants

To verify which gene(s) would be introduced into *T. reesei* and, therefore, be responsible for increased cellulase production, we randomly selected five transformants for genomic DNA extraction and determination of gene integration in the genome for the first round of transformation. The fungal genomic DNAs were isolated from the mycelia using a Fungal DNA kit (Omega Bio-Tek, Norcross, Georgia) and used as the template for PCR. Then, gene-specific primers (Additional file 1: Table S1) was designed and used to amplify the gene expressing cassettes from the transformants.

### Reverse transcription quantitative PCR

The transcript level of major cellulase, hemicellulase, and selected genes was measured by reverse transcription quantitative PCR (RT-qPCR). The mycelia of *T. reesei* were collected and pulverized at the maximum speed for 60 s in liquid nitrogen using a Mini-Beadbeater (Biospec, Bartlesville, USA). Total RNA was extracted using the TRIzol reagent (Thermo Fisher Scientific, Waltham, MA). The RNA (1 μg) was treated with DNase I and then reverse-transcribed to cDNA using the First Strand cDNA Maxima Synthesis kit (TOYOBO, Shanghai, China). RT-qPCR was performed in a QuantStudio 6 Flex Real-Time PCR System (Applied Biosystems, San Diego, CA) using a TransScript Green One-Step qRT-PCR SuperMix (TransGen, Beijing, China). The *actin* gene was used as a reference. The primers in RT-qPCR were listed in (Additional file 1: Table S1). The following PCR procedure was used: initial denaturation at 95ºC for 10 min and then 40 cycles of 94 ºC for 10 s, 60 ºC for 20 s, and 72 ºC for 30 s.

## Supplementary Information

**Additional file 1: Table S1**. Primers used in this study. **Figure S1**. Effect of overexpressing *xyr1* on cellulase production in SUS4. **Figure S2**. SDS-PAGE analysis of the fermentation broth of SUS4 and its mutant strains.

## Data Availability

All data supporting the conclusions of this article are included within the manuscript and Additional file 1.
