# Supplementary material for: Simultaneous manipulation of multiple genes within a same regulatory stage for iterative evolution of Trichoderma reesei
Source: Biotechnol Biofuels Bioprod. 2022 Mar 5;15:26. doi: 10.1186/s13068-022-02122-0 (PMC8898424; doi:10.1186/s13068-022-02122-0)
Supplement: Supplementary file 1 — Additional file 1: Table S1. Primers used in this study. Figure S1. Effect of overexpressing xyr1 on cellulase production in SUS4. Figure S2. SDS-PAGE analysis of the fermentation broth of SUS4 and its mutant strains. [file 13068_2022_2122_MOESM1_ESM.docx]

**Table S1. Primers used in this study**

| **Primer** | **Sequence 5'-3'** | **Usage** |
| --- | --- | --- |
| OriF1 | tttccataggctccgcccccctga | Amplification of Ampr-ori  Amplification of Ampr-ori |
| AmprR1 | ttctttgtttgaaaaattagcgct |  |
| Pcbh1F1 | ctaatttttcaaacaaagaatcaacctttggcgtttccctgatt | Amplification of *cbh1* promoter |
| Pcbh1R1 | ttaattaatacgtagtcgacgatgcgcagtccgcggttgactat | Amplification of *cbh1* promoter |
| Txyr1F | gtcgactacgtattaattaacatatgggaggccactcaatcgtatga | Amplification of *xyr1* terminator |
| Txyr1R | gggggcggagcctatggaaaacagccatgctcatcgtgcactt | Amplification of *xyr1* terminator |
| Xyr1F | gactgcgcatcgtcgactacgtaatgttgtccaatcctctccgtcgc | Amplification of *xyr1* |
| Ppdc1F1 | ctaatttttcaaacaaagaacgatgaaagccttgcaactgtgg | Amplification of *pdc1* promoter |
| Ppdc1R1 | ttaattaatacgtagtcgacgattgtgctgtagctgcgctgct | Amplification of *pdc1* promoter |
| Tpdc1F1 | gtcgactacgtattaattaacatatgcccggcatgaagtctgacc | Amplification of *pdc1* terminator |
| Tpdc1R1 | gggggcggagcctatggaaatcttaggagcggaatctgacatt | Amplification of *pdc1* terminator |
| Ace3F1 | agctacagcacaatcgtcgactacgtaatgctgcgctactcccccgtctta | Amplification of *ace3* |
| Ace3R1 | tcagacttcatgccgggcatatgttagccaacaacggtagtggacgt | Amplification of *ace3* |
| APAF1 | tacggttatccacagaatcagggg | Amplification of Ampr-ori-Ampr-pyr4 |
| APAR1 | ggatccgccaagcgcgcaattaac | Amplification of Ampr-ori-Ampr-pyr4 |
| Pgpd1pF1 | ggttaattgcgcgcttggcggatccctgcgcttcccacgcacagtacg | Amplification of *gpd1* promoter |
| Pgpd1pR1 | catatgttaattaatacgtagtcgactttgtatctgcgaattgagcttgc | Amplification of *gpd1* promoter |
| Peno1TF1 | gtcgactacgtattaattaacatatgatggccacgagagacaactacct | Amplification of *eno1* terminator |
| Peno1TR1 | gattctgtggataaccgtagaattcttctgtgtactctgtactttgac | Amplification of *eno1* terminator |
| 26163F | tcgcagatacaaagtcgactacgtacatgcctgcctcgagtgtcaacg | Amplification of *26163* |
| 26163R | tcgtggccatcatatgttaattaagttgtctcgtgttggaaatcttgg | Amplification of *26163* |
| 66966F | tcgcagatacaaagtcgactacgtaatggccaagaaggcgcgtcaaag | Amplification of *66966* |
| 66966R | tcgtggccatcatatgttaattaagctaggcgccgttgacgactccaa | Amplification of *66966* |
| 122523F | tcgcagatacaaagtcgactacgtaatggtagcacatagtctaccctct | Amplification of *122523* |
| 122523R | tcgtggccatcatatgttaattaagtcatatcggcaccatgtcgacgt | Amplification of *122523* |
| 80621F | tcgcagatacaaagtcgactacgtaatggctctcaacctcacctcgtc | Amplification of *80621* |
| 80621R | tcgtggccatcatatgttaattaagttagctgctctccacctgcttcag | Amplification of *80621* |
| PcDNA1pF1 | ggttaattgcgcgcttggcggatcccccgtaggacaagatcctgtcg | Amplification of *cDNA1* promoter |
| PcDNA1pR1 | catatgttaattaatacgtagtcgacgttgagagaagttgttggattg | Amplification of *cDNA1* promoter |
| PcDNA1tF1 | gtcgactacgtattaattaacatatgaagatcagactcttgtcgaggc | Amplification of *cDNA1* terminator |
| PcDNA1tR1 | gattctgtggataaccgtagaattcaaaaagacttgctatctacatcg | Amplification of *cDNA1* terminator |
| 80291F | acttctctcaacgtcgactacgtaatgggccggcaaccgagacaacg | Amplification of *80291* |
| 80291R | agtctgatcttcatatgttaattaagttatataaacggggcatcaatac | Amplification of *80291* |
| 64608F | acttctctcaacgtcgactacgtaagtgtcctaccaccccctccacg | Amplification of *64608* |
| 64608R | agtctgatcttcatatgttaattaagtcacgcagttgctgcctgaacgc | Amplification of *64608* |
| 53567F | acttctctcaacgtcgactacgtaatggctcccatcaccaaagagacc | Amplification of *53567* |
| 53567R | agtctgatcttcatatgttaattaagtcatttcatcgtcaccagctcct | Amplification of *53567* |
| 75769F | acttctctcaacgtcgactacgtaatggaccacacccagccgtaagt | Amplification of *75769* |
| 75769R | agtctgatcttcatatgttaattaagttacaacttggtgggagcggcag | Amplification of *75769* |
| 72685F | acttctctcaacgtcgactacgtaatgtctggccctgtgtaagtcggc | Amplification of *72685* |
| 72685R | agtctgatcttcatatgttaattaagttatgcctggtacgttgaggcgga | Amplification of *72685* |
| Pxpp1pF1 | ggttaattgcgcgcttggcggatccgtatcgtggtgtccagactatattg | Amplification of *xpp1* promoter |
| Pxpp1pR1 | catatgttaattaatacgtagtcgactgctgtatgggtaggttgaagg | Amplification of *xpp1* promoter |
| Pxpp1tF1 | gtcgactacgtattaattaacatatgagagagagagagagaggccagtgg | Amplification of *xpp1* terminator |
| Pxpp1tR1 | gattctgtggataaccgtagaattctcggggggaagaagattagaggag | Amplification of *xpp1* terminator |
| 123668F | acccatacagcagtcgactacgtaatgcctctcgttgtcgtcccagc | Amplification of *123668* |
| 123668R | tctctctctctcatatgttaattaagttaattgagcagcggctcgcgaa | Amplification of *123668* |
| 27600F | acccatacagcagtcgactacgtacgcccggcagcccgcggcaccgcc | Amplification of *27600* |
| 27600R | tctctctctctcatatgttaattaaggagctctcgctccagggttgatc | Amplification of *27600* |
| 74765F | acccatacagcagtcgactacgtaatgacttctgaagccccctctca | Amplification of *74765* |
| 74765R | tctctctctctcatatgttaattaagctactcgccctcttcgcctccca | Amplification of *74765* |
| 57940F | acccatacagcagtcgactacgtaatgtgctcgtcttacctctttcac | Amplification of *57940* |
| 57940R | tctctctctctcatatgttaattaagtcagatgacctcctctcgctcgt | Amplification of *57940* |
| PpkipF1 | ggttaattgcgcgcttggcggatcccgtggcagcacgagataacggtg | Amplification of *pki* promoter to construct the pPpki-Tcbh2 intermediate plasmid |
| PpkipR1 | catatgttaattaatacgtagtcgacggttaagagggttcttccggcttc | Amplification of *pki* promoter to construct the pPpki-Tcbh2 intermediate plasmid |
| PpkitF1 | gtcgactacgtattaattaacatatgatgcatggctttcgtgaccgggc | Amplification of *pki* terminator to construct the pPpki-Tcbh2 intermediate plasmid |
| PpkitR1 | gattctgtggataaccgtagaattctggtttccacgtgcacttacatgt | Amplification of *pki* terminator to construct the pPpki-Tcbh2 intermediate plasmid |
| 73903F | accctcttaaccgtcgactacgtaatgcccaagacacagcccaatct | Amplification of *73903* |
| 73903R | aagccatgcatcatatgttaattaagttacaagtatgccctccgagggaa | Amplification of *73903* |
| 52055F | accctcttaaccgtcgactacgtaatggccgcccgcaacatggccac | Amplification of *52055* |
| 52055R | aagccatgcatcatatgttaattaagttattgcttcagcgtggccttca | Amplification of *52055* |
| Xpp1PF2 | gtcgactacgtattaattaacatatgtgctgtatgggtaggttgaaggaa | Amplification of *xpp1* promoter to construct the cDNA1P-xpp1P intermediate plasmid |
| Xpp1PR2 | gattctgtggataaccgtagaattcgtatcgtggtgtccagactatat | Amplification of *xpp1* promoter to construct the cDNA1P-xpp1P intermediate plasmid |
| Cre1iF1 | acttctctcaacgtcgactacgtaatgcaacgagcacagtctgccgtg | Amplification of *cre1* RNAi fragment |
| Cre1iR1 | cccatacagcacatatgttaattaagaggcatgacaaaggacgagtagga | Amplification of *cre1* RNAi fragment |
| Ace1iF1 | acttctctcaacgtcgactacgtagtcccggaaccgactgcgaacatg | Amplification of *ace1* RNAi fragment |
| Ace1iR1 | cccatacagcacatatgttaattaagagagtcggcggcgccagccttgtc | Amplification of *ace1* RNAi fragment |
| Yps1iF1 | acttctctcaacgtcgactacgtagatacctttgacgaggtcggccaa | Amplification of *yps1* RNAi fragment |
| Yps1iR1 | cccatacagcacatatgttaattaagatcctcgcttgtggagagcgtgtc | Amplification of *yps1* RNAi fragment |
| Ptef1pF1 | ggttaattgcgcgcttggcggatcctcgaatgtgactgcacacaccaca | Amplification of *tef1* promoter to construct the Ptef1-Ppki intermediate plasmid |
| Ptef1pR1 | catatgttaattaatacgtagtcgactttgacggtttgtgtgatgtagcg | Amplification of *tef1* promoter to construct the Ptef1-Ppki intermediate plasmid |
| PpkipF2 | gtcgactacgtattaattaacatatgggttaagagggttcttccggcttc | Amplification of *pki* promoter to construct the Ptef1-Ppki intermediate plasmid |
| PpkipR2 | gattctgtggataaccgtagaattccgtggcagcacgagataacggtga | Amplification of *pki* promoter to construct the Ptef1-Ppki intermediate plasmid |
| Ymr1iF1 | caaaccgtcaaagtcgactacgtaatcatcgaacatgtccaggccatc | Amplification of *ymr1* RNAi fragment |
| Ymr1iR1 | accctcttaacccatatgttaattaagctggggtcatagatgttccagcc | Amplification of *ymr1* RNAi fragment |
| Och1-2iF1 | caaaccgtcaaagtcgactacgtaaccctgatcggccagagaggaggc | Amplification of *och1-2* RNAi fragment |
| Och1-2iR1 | accctcttaacccatatgttaattaagagttcatcacctcgaagctctccg | Amplification of *och1-2* RNAi fragment |
| Pep4iF1 | caaaccgtcaaagtcgactacgtatcgttcaaggttgtcctcgacacg | Amplification of *pep4* RNAi fragment |
| Pep4iR1 | accctcttaacccatatgttaattaagtactcaatcttgccctcgtagtga | Amplification of *pep4* RNAi fragment |
| doa10iF1 | caaaccgtcaaagtcgactacgtaaacatcgagactcgacccgacac | Amplification of *doa10* RNAi fragment |
| doa10iR1 | accctcttaacccatatgttaattaagggacccggtagccaggccctcgtc | Amplification of *doa10* RNAi fragment |
| Ppdc1pF2 | ggttaattgcgcgcttggcggatcccgatgaaagccttgcaactgtggt | Amplification of *pdc1* promoter to construct the Ppdc1-Peno1 intermediate plasmid |
| Ppdc1pR2 | catatgttaattaatacgtagtcgacgattgtgctgtagctgcgctgct | Amplification of *pdc1* promoter to construct the Ppdc1-Peno1intermediate plasmid |
| Peno1pF2 | gtcgactacgtattaattaacatatgtttgaagctatttcaggtggctgg | Amplification of *eno1* promoter to construct the Ppdc1-Peno1 intermediate plasmid |
| Peno1pR2 | gattctgtggataaccgtagaattcttctcaaataccgcagaggcgac | Amplification of *peno1* promoter to construct the Ppdc1-Peno1intermediate plasmid |
| Sed1iF1 | gcacaatcgtcgactacgtattaattaacatgaagttcaccgtcgctgtcgct | Amplification of *sed1* RNAi fragment |
| Sed1iR1 | cctgaaatagcttcaaacatatggccagtggggacggcgggaggagc | Amplification of *sed1*RNAi fragment |
| Ych1iF1 | gcacaatcgtcgactacgtattaattaactgactattgcgtcgctcaagaga | Amplification of *ych1* RNAi fragment |
| Ych1iR1 | cctgaaatagcttcaaacatatgctccaaatctccctccgaaacc | Amplification of *ych1* RNAi fragment |
| Der1iF1 | gcacaatcgtcgactacgtattaattaacatggcagagctttccacggatgcg | Amplification of *der1* RNAi fragment |
| Der1iR1 | cctgaaatagcttcaaacatatgcgaagtgcaggggtaatctccctc | Amplification of *der1* RNAi fragment |
| YPcbh1F | caagaacaatagccgataaagat | Verification of chromosomal integration of *xyr1* |
| Yxyr1R | agagccctggcgctgctttggc | Verification of chromosomal integration of *xyr1* |
| Yace3F | tcctgattatgttgtctggatgctg | Verification of chromosomal integration of *ace3* |
| YPdc1TR | cctctcatgcgacgtgcaagatta | Verification of chromosomal integration of *ace3* |
| YEno1TR | cacgcagcggtgtctgtcaagcta | Verification of the joint sequence of *26163* /*66966* /*122523* and Eno1T |
| Y26163F | gtttctgtatcaagcagccatgata | Verification of chromosomal integration of *26163* |
| Y66966F | gcaatgaccagctgcttcacccaa | Verification of chromosomal integration of *66966* |
| Y122523F | agttcaaccagtcggcgccttcag | Verification of chromosomal integration of *122523* |
| YcDNA1TR | gtcaactgcacaatttgcgtgtaa | Verification of chromosomal integration of of *80291*/64608 |
| Y80291F | gagttgtccatccagattctcaga | Verification of chromosomal integration of *80291* |
| Y64608F | agaacggacaccaacacctcggc | Verification of chromosomal integration of *64608* |
| YXpp1TR | cccatacaaacaattaaatcaatcc | Verification of the joint sequence of *123668*/*27600*/*74765* and Xpp1T |
| Y123668F | actgacccggaccatcaaagacgc | Verification of chromosomal integration of *123668* |
| Y27600F | ccatcatcgacatcatgggagcatg | Verification of chromosomal integration of *27600* |
| Y74765F | cctcagcacagtgaagatgaagat | Verification of chromosomal integration of *74765* |
| YcDNA1PF | ataatgcttgggttggaagcgattc | Verification of chromosomal integration of *cre1* |
| Yxpp1PR | actctaagacggcagtcggagcat | Verification of chromosomal integration of *ace1* |
| actF | tgagagcggtggtatccacg | RT-qPCR for *actin* |
| actR | ggtaccaccagacatgacaatgttg | RT-qPCR for *actin* |
| qcbh1F | gctgccggtgcggcttgaac | RT-qPCR for *cbh1* |
| qcbh1R | ctggccattgatgaacttcagatcgc | RT-qPCR for *cbh1* |
| qcbh2F | atcgcaacatttggaaggttcag | RT-qPCR for *cbh2* |
| qcbh2R | ctggccattgatgaacttcagatcgc | RT-qPCR for *cbh2* |
| qegl1F | gactacacggaggagctcgac | RT-qPCR for *egl1* |
| qegl1R | ttgcgagtagtagtcgttgctata | RT-qPCR for *egl1* |
| qegl2F | gtaccacagatggcacttgc | RT-qPCR for *egl2* |
| qegl2R | agagacaggcacccctgaac | RT-qPCR for *egl2* |
| qxyr1F | agcgccagggctctcttatt | RT-qPCR for *xyr1* |
| qxyr1R | gcatccaaggccgaattct | RT-qPCR for *xyr1* |
| qace3F | tcgtttccttcctcggcact | RT-qPCR for *ace3* |
| qace3R | gtgaatcctggttgcgatgg | RT-qPCR for *ace3* |
| q66966F | agcagtggcgccctggtgct | RT-qPCR for *66966* |
| q66966R | gccaaggctcactctctgatca | RT-qPCR for *66966* |
| q27600F | gcgccacgtgcattcagtcc | RT-qPCR for *27600* |
| q27600R | tcgtccgggtgcgatggcggc | RT-qPCR for *27600* |
| q74765F | atgacttctgaagccccctctca | RT-qPCR for *74765* |
| q74765R | gatgcggttgagcccatgaga | RT-qPCR for *74765* |
| q26163F | ggagaatgtagaatacgactctg | RT-qPCR for *26163* |
| q26163R | ggctatcaagatgatgtcgat | RT-qPCR for *26163* |
| q64608F | agtgtcctaccaccccctccacg | RT-qPCR for *64608* |
| q64608R | acatatgttccctctcccacca | RT-qPCR for *64608* |
| qder1F | gctggaaacatggttctgggag | RT-qPCR for *der1* |
| qder1R | agtcgccagtactgtgacttga | RT-qPCR for *der1* |
| qYCH1F | gcgggatgtcgactacataggc | RT-qPCR for *ych1* |
| qYCH1R | gctgctggctcagcgcgcagtgg | RT-qPCR for *ych1* |
| qsed1F | gctacacctgcggtgctgct | RT-qPCR for *sed1* |
| qsed1R | accaccagtgctggcgggagg | RT-qPCR for *sed1* |
| qYMR1F | aagccaagaagatcatcgaaca | RT-qPCR for *ymr1* |
| qYMR1R | aggcgtcgactcttggccgtct | RT-qPCR for *ymr1* |
| qOCH1-2F | gcagattgtgctgcccaagg | RT-qPCR for *och1-2* |
| qOCH1-2R | ccgatcagggtgtactgccagtc | RT-qPCR for *och1-2* |
| qpep4F | agcagcagctggagggttcgag | RT-qPCR for *pep4* |
| qpep4R | cattcatgaagttggtgacgg | RT-qPCR for *pep4* |
| qdoa10F | ggtgtcttgtacttcatccga | RT-qPCR for *doa10* |
| qdoa10R | cacaagtgctccataaacgaatg | RT-qPCR for *doa10* |
| qyps1F | attcggaagcttcaaccctgat | RT-qPCR for *yps1* |
| qyps1R | ccatggtgagattcttgacggt | RT-qPCR for *yps1* |
| q80621F | ctcttcacgggacttgccgccc | RT-qPCR for *80621* |
| q80621R | ttgcgcagaacggcgtgtccgta | RT-qPCR for *80621* |
| q75759F | cttcggcctgtaccgaaacca | RT-qPCR for *75759* |
| q75759R | gaactcctggagctgctgctca | RT-qPCR for *75759* |
| q535675F | caatgttggatgcgtccccaa | RT-qPCR for *53567* |
| q53567R | atgtacgcgtcgcgcttggtct | RT-qPCR for *53567* |
| q52055F | cctcaagcagatgtggctctct | RT-qPCR for *52055* |
| q52055R | aaaggcgtggcggccgatgat | RT-qPCR for *52055* |
| q73903F | atgcccaagacacagcccaatc | RT-qPCR for *73903* |
| q73903R | ccgcccgagacggccaccttga | RT-qPCR for *73903* |
| q57940F | attgccgtccagtactggcgca | RT-qPCR for *57940* |
| q57940R | gatcctccttctggttgaggt | RT-qPCR for *57940* |
| q72685F | cttgctgtcatgggacaga | RT-qPCR for *72685* |
| q72685R | cgacaatagacttgcccttggc | RT-qPCR for *72685* |


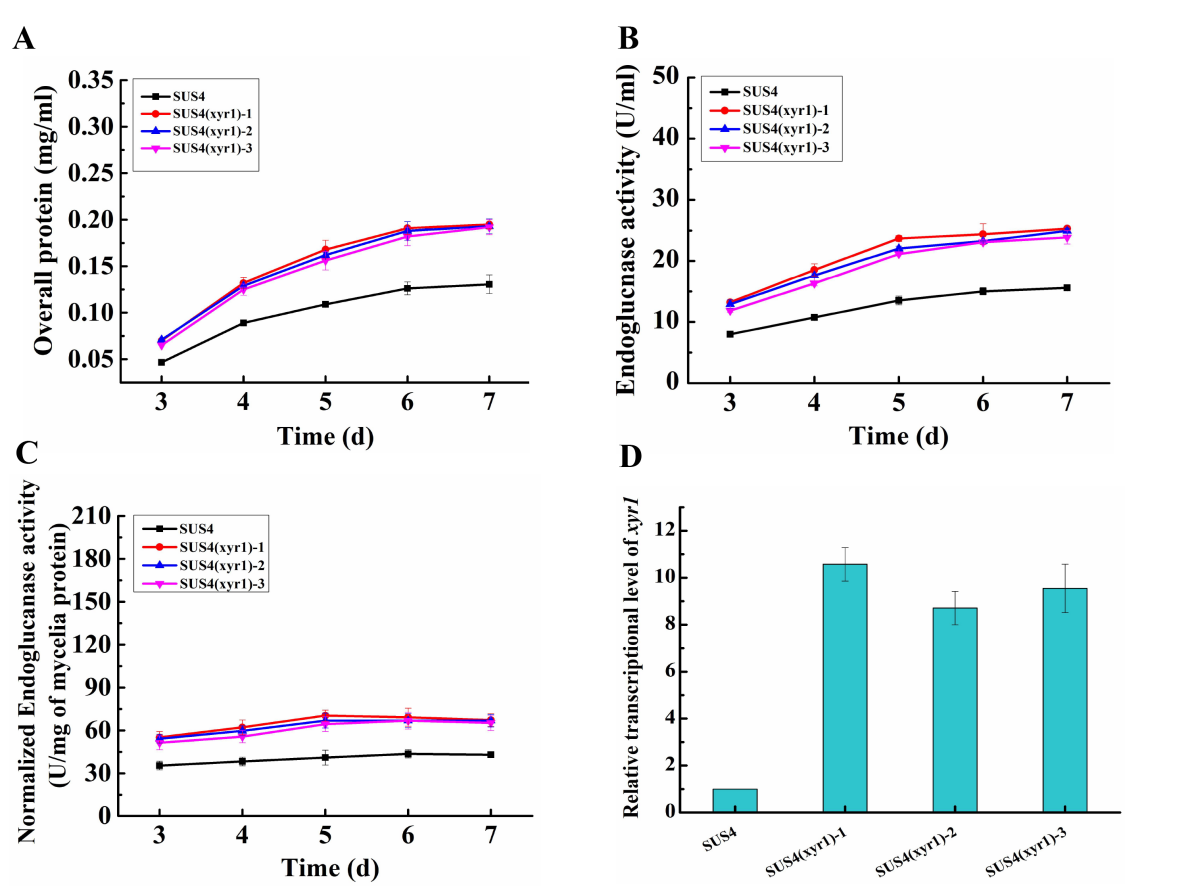


**Figure S1.** Effect of overexpressing *xyr1* on cellulase production in SUS4. A. Extracellular overall protein concentration; B. Endoglucanase activity; C. Normalized endoglucanase activity against mycelial biomass. D. The transcriptional level of *xyr1* at 24 h post cellulose induction.

**
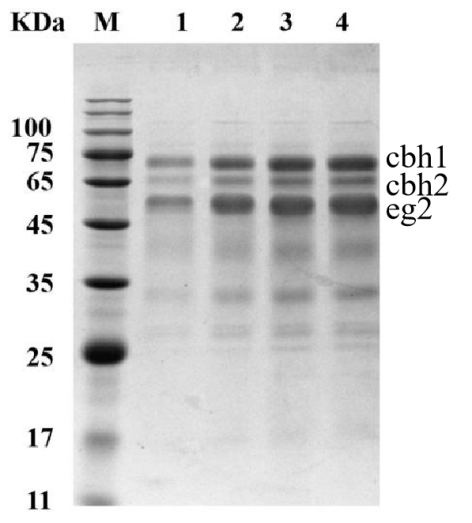
**

**Figure S2.** SDS-PAGE analysis of the fermentation broth of SUS4 and its mutant strains. M: protein molecular mass marker; lane 1: the parent strain SUS4; lane 2: strain 1-2; lane 3: strain 2-3; lane 4: strain 3-8. Ten μl of the fermentation supernatant for each representative transformant, which was collected on day 5 post cellulase induction, were used for SDS-PAGE analysis.

.
